# Female Economic Dependence and the Morality of Promiscuity

**DOI:** 10.1007/s10508-014-0320-4

**Published:** 2014-06-25

**Authors:** Michael E. Price, Nicholas Pound, Isabel M. Scott

**Affiliations:** Department of Psychology, School of Social Sciences, Brunel University, Uxbridge, Middlesex UB8 3PH UK

**Keywords:** Promiscuity, Sociosexuality, Paternity certainty, Parental investment, Evolutionary moral psychology

## Abstract

In environments in which female economic dependence on a male mate is higher, male parental investment is more essential. In such environments, therefore, both sexes should value paternity certainty more and thus object more to promiscuity (because promiscuity undermines paternity certainty). We tested this theory of anti-promiscuity morality in two studies (*N* = 656 and *N* = 4,626) using U.S. samples. In both, we examined whether opposition to promiscuity was higher among people who perceived greater female economic dependence in their social network. In Study 2, we also tested whether economic indicators of female economic dependence (e.g., female income, welfare availability) predicted anti-promiscuity morality at the state level. Results from both studies supported the proposed theory. At the individual level, perceived female economic dependence explained significant variance in anti-promiscuity morality, even after controlling for variance explained by age, sex, religiosity, political conservatism, and the anti-promiscuity views of geographical neighbors. At the state level, median female income was strongly negatively related to anti-promiscuity morality and this relationship was fully mediated by perceived female economic dependence. These results were consistent with the view that anti-promiscuity beliefs may function to promote paternity certainty in circumstances where male parental investment is particularly important.

## Introduction

Beliefs about sexual morality are a powerful cultural force in many societies. In the U.S., for example, diverging views on issues such as abortion, gay marriage, and sex education seem influenced by differing beliefs, often religion-related, about sexual morality. Research on the link between religion and sexual morality has viewed religiosity as an extension of a long-term monogamous mating strategy (Weeden, Cohen, & Kenrick, 2008) or as an effort to promote paternity certainty (Strassman et al., 2012). Much remains to be investigated, however, about whether particular forms of sexual morality are likely to emerge in some environments more than others, perhaps as solutions to specific adaptive problems faced by individuals in those environments. In this article, we report two studies which tested predictions about U.S. residents’ moral views on promiscuous mating, using a theory which regards these views as facultative solutions to adaptive problems related to promoting paternity certainty and conforming to local social norms. Previous researchers have attempted to explain why different types of mating systems/behaviors (e.g., monogamy versus polygyny or promiscuity) have emerged in different human societies, often with an emphasis on the role of environment and parental investment (Fortunato & Archetti, 2010; Gavrilets, 2012; Henrich, Boyd, & Richerson, 2012; Schmitt, 2005a) and our research was, in general, complementary to these approaches. However, our work is unique in many of its predictions and in its focus on moral attitudes about promiscuity.

### Short-Term and Long-Term Mating Across Species and Cultures

A variety of different mating strategies exist across animal species and human cultures. The terms most commonly used by biologists and anthropologists (e.g., Clutton-Brock, 1989; Murdock, 1967; Schmitt, 2005b) to classify mating strategies include monogamous (one male mates with one female over an extended period, such as over one or more breeding seasons), polygynous (one male mates with multiple females over an extended period), polyandrous (one female mates with multiple males over an extended period), and promiscuous (or “multimale-multifemale”; multiple females engage in short-term, non-exclusive relationships with multiple males). However, the mating behaviors of a given species, culture, or individual may be complex and “strategically pluralistic” (Gangestad & Simpson, 2000), that is, characterized by more than one strategy. For example, consider the mating systems of humans’ closest evolutionary relatives, the greater and lesser apes. Although most commonly chimpanzees and bonobos are classified as promiscuous, gorillas and orangutans as polygynous, and gibbons as monogamous (Schmitt, 2005b; Smuts & Smuts, 1993), these categories may mask considerable strategic pluralism. For example, monogamous gibbons sometimes engage in short-term extrapair copulations and polygynous orangutans are often also considered promiscuous (Beaudrot, Kahlenberg, & Marshall, 2009; Plavcan, 2012).

Human mating systems can also exhibit considerable levels of strategic pluralism. Anthropologists have classified more than 80 % of preindustrial societies as polygynous, 16 % as monogamous, and less than 1 % as polyandrous (Murdock, 1967; Schmitt, 2005b). Yet, within most “polygynous” societies, most long-term relationships are, in fact, monogamous; the polygynous label indicates only that polygyny is permitted and commonly observed (Stewart-Williams & Thomas, 2013). Further, although these categories focus on long-term mating, short-term strategies are also frequently observed in these societies. For example, anthropologists estimate that extramarital sex occurs at least “occasionally” among males in 80 % and among females in 73 % of preindustrial cultures and that comparable rates for premarital sex are 88 % for males and 80 % for females. Further, wife sharing is estimated to occur in 39 % of these cultures (Broude & Greene, 1976; Schmitt, 2005b). Such within-culture co-existence of long-term and short-term mating strategies is also evident in industrialized societies. In the U.S. and other wealthy democracies, for instance, although long-term monogamy is common, so are short-term sexual relationships (Chandra, Mosher, Copen, & Sionean, 2011). However, many Americans—particularly those who are strongly religious and/or politically conservative—object morally to short-term mating and believe that promiscuity is wrong (Klein, 2012).

### The Evolution of Sexual Strategic Pluralism in Humans

Cross-culturally and on average, men exhibit greater motivation than women to engage in short-term mating (Schmitt, 2005a), which is consistent with the fact that they, as the sex with lower obligatory parental investment, can generally derive more reproductive benefits from having many mates (Trivers, 1972). However, although men can benefit from short-term mating under a wider range of circumstances than can women, in ancestral environments, a willingness to mate with multiple males under certain circumstances (i.e., facultative polyandry) could potentially have benefited females in several ways (Greiling & Buss, 2000; Smith, 1984). For example, multiple matings could have facilitated resource acquisition, either in direct exchange for sex (Symons, 1979) or by eliciting paternal investment from multiple men via paternity confusion (Hrdy, 1981). Additionally, indirect benefits may have been derived by ancestral women who accepted resources and parental effort from a primary mate while engaging in extra-pair copulations with men of superior genetic quality (Gangestad & Thornhill, 2008; Greiling & Buss, 2000; Smith, 1984). Extra-pair sex may also have served as a useful “insurance” against the possibility of infertility in a primary mate or as a means to promote genetic diversity in offspring as a “hedge” against environmental unpredictability (Smith, 1984). Potential genetic benefits of multiple mating for females are reviewed comprehensively by Jennions and Petrie (2000).

Women vary substantially in their willingness to engage in short-term mating (Simpson & Gangestad, 1991) and evidence suggests that some of this variation reflects females making trade-offs between producing offspring of “high genetic quality” and securing male parental investment (Gangestad & Simpson, 2000). Across species, in those where male parental investment is very low, relationships tend to be short-term and female mate choice tends to reflect “good genes” sexual selection; that is, females choose males based more on signals of heritable qualities than on “good provider” criteria (i.e., value as a source of investment). In species where male parental investment is more vital, however, female choice tends to be based more on good provider criteria (Gangestad & Simpson, 2000; Schmitt, 2005a). Some species exhibit a mix of both strategies (Gangestad, 2000) and human mating behavior appears to be an example of such strategic pluralism: females base mate choices flexibly on both good genes and good provider criteria, with the importance of each kind of criteria varying facultatively according to female characteristics and context (Gangestad & Simpson, 2000). As such, women are expected to pursue some kinds of short-term mating opportunities; for example, in some contexts to mate with a man whose genetic quality is high enough to sufficiently offset the risk that he would be a poor provider. However, when dependence on male parental investment is greater, females should be less inclined to choose males based solely on short-term, good genes criteria.

If short-term mating is less common when females depend more on male parental investment and if females depend more on male parental investment in harsher environments, then short-term mating should be less common in those environments (Gangestad & Simpson, 2000). Schmitt (2005a), drawing on data collected from a cross-national sample (*N*s ranging from 20 to 48), provided evidence to support this hypothesis: national indicators of ecological/economic hardship (e.g., child malnutrition, life expectancy, gross domestic product) correlated moderately-to-strongly negatively with male and, especially, female interest in short-term mating, i.e., national mean sociosexuality scores (Simpson & Gangestad, 1991). Schmitt (2005a) also found national sociosexuality scores to be strongly negatively related to national operational sex ratio (ratio of males to females of reproductive age), a result consistent with sex ratio theory (Pedersen, 1991). According to this theory, short-term strategies should be more common in countries with lower operational sex ratio, because, as noted above, men are relatively interested in short-term mating. When men are relatively scarce, their bargaining power on the mating market increases, which should help them pursue short-term relationships.

### Female Economic Dependence on a Male Mate as a Predictor of Anti-Promiscuity Morality

In order for a man’s parental investment to benefit his offspring, he must know who his offspring are and establishing paternity was probably a major adaptive problem for ancestral humans (Daly, Wilson, & Weghorst, 1982; Symons, 1979). An ancestral male could have benefited by facultatively adjusting his level of investment in a woman and her offspring according to the probability that her offspring were also his own (Gray & Anderson, 2010), by investing more in a mate when he had greater confidence in her sexual fidelity. Accordingly, evidence suggests that men have evolved emotional and behavioral responses to female infidelity that ancestrally would have reduced both the risk and the costs of cuckoldry (Daly et al., 1982).

Since a man can adjust his investment in a mate and/or her offspring based on his likelihood of being (or becoming) the father of her offspring, men and women should be more averse to promiscuity when females depend more on male parental investment. This increased aversion should occur, in part, because the costs of promiscuity—to both mated females who seek male parental investment and mated males who seek to provide it—will increase with female dependence on male parental investment. When a female and her offspring depend more on male investment, this investment is more valuable to her, her offspring, and the male providing it (if the offspring are also his own). Further, when females depend more on this investment, it should also be costlier for males to provide, because its increased value should motivate men to expend more time and energy to produce it. Due to the increased value and cost of male parental investment under conditions of greater female dependence, actions which undermine paternity certainty (and which thus reduce male motivation to produce parental investment), such as promiscuity, will become more threatening to both mated men and mated women. As outlined in Table 1, this includes promiscuity by one’s self, by one’s mate, and by one’s same-sex reproductive competitors. Moreover, when female dependence is higher, not only do the costs of promiscuity go up, but the benefits of promiscuity go down, for both sexes. This is true because when male parental investment is more valuable (1) females are less able to reproduce successfully with “good genes” but low-investing males and (2) males are less able to reproduce successfully via low-investment strategies.

**Table 1:** Reasons why promiscuity by self and others becomes costlier (to mated individuals of either sex) in environments in which female economic dependence on a male mate is higher

	Whose promiscuity is threatening?	
	Own	Mate’s or same-sex competitors’
Who does this promiscuity threaten?		
Females	Greater desertion costs, so own promiscuity may trigger costlier desertion by mate; greater cuckoldry costs, so own promiscuity may trigger harsher retaliation by mate	Greater desertion costs, so mate’s promiscuity (with same-sex competitors) may lead to costlier desertion
Males	Greater desertion costs, so own promiscuity may trigger harsher retaliation by mate	Greater costs of producing mating/parental investment, so being cuckolded by mate (and same-sex competitors) involves costlier waste of investment

The theory presented here, then, predicts that both sexes should be more averse to promiscuity in environments characterized by greater female economic dependence on a male mate. We will refer to this theory as the female economic dependence theory of promiscuity aversion and expect this aversion to manifest itself as greater willingness to express moral disapproval of promiscuity. Through moralizing, individuals can promote behavior which serves their own personal and coalitional interests and, when more (powerful) people in a society have an interest in discouraging a behavior, their moral system will more likely proscribe that behavior (Alexander, 1987; Price, Kang, Dunn, & Hopkins, 2011).

### The Current Studies

Using U.S. samples, we tested predictions of the female economic dependence theory at both the individual and state levels. Specifically, we tested whether opposition to promiscuity was higher among (1) individuals who perceived female economic dependence on a male mate to be relatively high in their social network and (2) individuals who were themselves currently (or likely to someday be) in a heterosexual relationship involving relatively high female economic dependence. We also examined state-level economic indicators (e.g., female income, availability of welfare benefits) related to female economic dependence in order to test (3) whether indicators of greater female dependence relate positively to anti-promiscuity morality and (4) whether any such relationships are mediated by the extent of perceived female economic dependence in one’s social network. Finally, we tested the predictions that opposition to promiscuity would be higher (5) among females than among males, as predicted by the sex differences theory and (6) in states with higher male–female sex ratios, as predicted by the sex ratio theory.

We expected that environments characterized by greater female economic dependence would tend to generate anti-promiscuity moral systems which, like all moral systems, impose social costs on norm violators (Ostrom, 2000; Price, 2005, 2006). Such costs should incentivize group members to adopt the norms about promiscuity which prevail in their social network, regardless of personal economic circumstances. Therefore, the predictor of anti-promiscuity morality of primary interest was perceived female economic dependence among females in one’s social network. However, in Study 2, we examined the role of personal circumstances as well, considering the predictive utility of extent of one’s personal involvement, or likelihood of being involved, in a relationship involving high female economic dependence (based on reported income of one’s self and of one’s relationship partner).

We also examined the effects of several control variables on anti-promiscuity morality, including age, which could correlate with sexual conservatism and also with other predictors (e.g., income), as well as religiosity and political conservatism, which were expected to correlate positively with anti-promiscuity morality. We also controlled for the anti-promiscuity views of each participant’s nearest geographical neighbors. It is important to measure neighbors’ traits in cross-cultural comparative research, due to issues with non-independence that can arise from spatial proximity (spatial autocorrelation). Cultural traits may be transmitted, via common (cultural) ancestry, copying or borrowing, in “packages”. The dispersal of such packages can lead to a false impression of a causal or structural relationship between pairs of traits (Eff, 2008; Pagel & Mace, 2004) with associations between traits arising due to the dispersion of a single founding culture whose members shared those traits.

## Study 1

### Method

#### Participants

Participants (*N* = 656, 52.9 % male) were U.S. residents aged 18 to 80 years (*M* = 32.71, *SD* = 11.26). The sample was 81 % European American, 7 % African American, 7 % Asian American, 4 % Latino American, and 2 % other. All participants were recruited via Amazon.com’s MTurk, a crowdsourcing website that is widely used in scientific research (Paolacci, Chandler, & Ipeirotis, 2010). Although U.S. MTurk workers probably have some characteristics that distinguish them from the U.S. general population (e.g., a desire to earn extra money, an affinity for online tasks), they appear to be at least as representative of the U.S. population as other kinds of commonly-used samples, such as university student and standard internet samples (Paolacci et al., 2010). The quality of psychological data collected via MTurk tends to be high, and comparable to more traditional data collection methods, in terms of psychometric standards such as internal consistency and test–retest reliability (Buhrmester, Kwang, & Gosling, 2011). Further, the results of studies conducted over MTurk tend to be highly comparable to those conducted using other kinds of samples (Crump, McDonnell, & Gureckis, 2013; Paolacci et al., 2010).

#### Procedure

A notice was posted on MTurk offering U.S. residents aged 18 years and above US$1.00 to complete an online “Relationship Attitudes” survey. MTurk workers were provided with a link to the survey, and after indicating their informed consent and completing the survey, they were compensated via MTurk. All data were collected from 23 to 26 April, 2012.

#### Measures

Perceived female economic dependence was composed of eight items (Cronbach’s α = .94), such as “Most women I know depend heavily on the money of a male partner, or probably will at some point in their life.” Wrongness of promiscuity was composed of 12 items (Cronbach’s α = .96), such as “Promiscuous (men/women) are not worthy of much respect”; six statements referred to male promiscuity and six to female promiscuity. Perceived female economic dependence and wrongness of promiscuity were composite variables, scored as the mean response on a 7-point scale from “Disagree strongly” to “Agree strongly.” Religiosity was the summed *z*-scores of responses to the five items measuring religious commitment (Cronbach’s α = .90) from Kurzban, Dukes, and Weeden (2011). Political conservatism was the response to the item “How would you describe yourself politically, on a 1–5 scale of liberal to conservative?”, on a 5-point scale from “very liberal” to “very conservative.” For all items from all Study 1 composite variables, see Appendix A.

### Results

Descriptive statistics and intercorrelations are shown in Table 2. The correlation between perceived female economic dependence and wrongness of promiscuity was significant, positive, and of moderate size, *r*(654) = .28, *p* < .001; perceptions of high female economic dependence were associated with perceptions that promiscuity is wrong. A linear regression model was created with wrongness of promiscuity as the outcome variable and sex (males coded as 0, females as 1), female economic dependence, religiosity, and conservatism as predictors. All predictors except age produced significant beta coefficients (Table 3). Because these coefficients are standardized, they can be used to rank predictors in terms of their effect on wrongness of promiscuity. (Beta coefficients in Table 3, for example, show that religiosity had the strongest effect of all predictors). When this analysis was conducted as a hierarchical regression, with perceived female economic dependence entered on the second step after the other four predictors had been entered on the first step, perceived female economic dependence explained an additional 2 % of variance in wrongness of promiscuity (Δ*R* ^2^ = .02, *F* = 20.68, *p* < .001).

**Table 2:** Intercorrelations, means, and *SD*s for Study 1 variables

Variable	1	2	3	4	5	*M*	*SD*	*N*
1. Age	–	.07	.20**	.08	.14*	35.44	11.61	310
2. Perceived FED	.05	–	.15**	.23***	.27***	3.95	1.45	309
3. Religiosity	.14**	.25***	–	.44***	.55***	0.15	0.85	309
4. Conservatism	.09	.22***	.37***	–	.44***	2.52	1.10	309
5. Wrongness of promiscuity	.07	.30***	.54***	.36***	–	3.51	1.72	309
*M*	30.27	4.08	−0.13	2.66	2.94			
*SD*	10.36	1.37	0.81	1.07	1.55			
*N*	347	347	348	348	346			

Intercorrelations for males are below the diagonal, and intercorrelations for females are above the diagonal. Means and *SD*s for males are presented in the horizontal rows, and means and *SD*s for females are presented in the vertical columns. Perceived FED = perceived female economic dependence. * *p* < .05, ** *p* < .01, *** *p* < .001

**Table 3:** Linear regression of wrongness of promiscuity on Study 1 predictors

	*β*	*t*	*p*
Age	<.01	<1	ns
Sex (males = 0, females = 1)	.11	3.33	.001
Perceived female economic dependence	.14	4.44	<.001
Religiosity	.44	12.62	<.001
Conservatism	.19	5.37	<.001
	Overall: *N* = 650, total *R* = .61, Adj *R* ^2^ = .37, *p* < .001.		

We also conducted supplementary analyses in which this model shown in Table 3 was considered when the participants were males only or females only and when the outcome variable was opposition to male promiscuity only or opposition to female promiscuity only. In all of these cases, the same general patterns were observed as those shown in Table 3.

### Discussion

Study 1 results showed that perceived female economic dependence was moderately predictive of opposition to promiscuity and this relationship remained significant after controlling for the effects of age, sex, religiosity, and conservatism. Results also indicated that women, on average, were more opposed to promiscuity than men. Study 2 aimed to (1) replicate these results, while adding another control variable: the anti-promiscuity views of one’s geographical neighbors; (2) investigate how well opposition to promiscuity was predicted by one’s own involvement (or likely involvement) in a relationship entailing high female economic dependence; and (3) collect enough data from across the U.S. to determine (1) which state-level economic indicators were most related to perceived female economic dependence and to anti-promiscuity morality and (2) whether perceived female economic dependence mediated any relationships that may exist between economic indicators and anti-promiscuity morality.

## Study 2

### Method

#### Participants

As in Study 1, Participants (*N* = 4,626, 51.9 % male) were U.S. MTurk workers aged 18–80 years (*M* = 28.88, *SD* = 10.22). They were 78 % European American, 9 % Asian American, 6 % African-American, 5 % Latino-American, and 2 % other. Regarding sexual orientation, 90 % were heterosexual, 6 % bisexual, and 3 % homosexual.

A total of 4,533 of 4,626 participants provided a valid ZIP code that was consistent with provided city and state names and were included in subsequent analyses. Approximate geographic coordinates (latitude and longitude) based on ZIP code centroids were determined using provided ZIP codes and the CivicSpace US ZIP Code Database (CivicSpace Labs, 2004). Participants came from all 50 states and the District of Columbia. The mean state *N* was 88.84 (*SD* = 103.34, range, 2–570), with a very high correlation (*r* = .99) between a state’s *N* and its 2011 population (U.S. Census Bureau, 2012a).

#### Procedure

A notice was posted on MTurk offering U.S. residents aged 18 years and above US$0.70 to complete an online “Relationship Attitudes” survey. MTurk workers were provided with a link to the survey, and after indicating their informed consent and completing the survey, they were compensated via MTurk. All survey data were collected from July 4–7, 2012.

#### Measures

##### Individual-Level Variables

Measures of perceived female economic dependence, wrongness of promiscuity, and religiosity were abbreviated from Study 1 versions. Details of abbreviation procedures and variable items are shown in Appendix B. Cronbach’s α was .91 for female economic dependence and .97 for wrongness of promiscuity. Political conservatism was measured as in Study 1. Income of self and (if applicable) partner was measured on a 13-point scale from “less than $10,000” to “more than $120,000.” Relationship status was recorded with the question “Do you currently live with a long-term romantic relationship partner (such as a spouse)?”, response choices were “yes” or “no.”

##### State-Level Data on Income, Sex Ratio, and Welfare Benefits

The U.S. Census Bureau’s 2011 American Community Survey provided state-level data on income and operational sex ratio. Income data were median earnings of people aged 16 years and above, over the 12 months preceding the survey (U.S. Census Bureau, 2012b). Operational sex ratio was based on estimated numbers of males and females aged 15–49 (following Schmitt [2005a]) in each state (U.S. Census Bureau, 2012a).

Welfare data measured the main sources of benefits available to women in each state. These included fiscal year 2011 expenditures on TANF (Temporary Assistance for Needy Families [U.S. Department of Health & Human Services, 2012]), SNAP (Supplemental Nutrition Assistance Program [U.S. Department of Agriculture, 2012a]), and WIC (Women, Infants and Children [U.S. Department of Agriculture, 2012b]). Summing these expenditures produced a total welfare amount for each state; on average, SNAP constituted 69 % of this total, TANF 27 %, and WIC 4 %. Totals were divided by the 2011 state female population (U.S. Census Bureau, 2012a) to obtain per-woman welfare spending by state.

##### Anti-Promiscuity Views of Nearest Neighbors

In order to control for the possibility of spatial autocorrelation (e.g., positive associations between the anti-promiscuity views of close geographical neighbors), we conducted a spatial lag analysis using OpenGeoDa 1.2.0 (GeoDa Center for Geospatial Analysis and Computation, Arizona State University). For each participant, using geographical coordinates inferred from ZIP code, all “neighbors” (i.e., other participants within a 100-mile radius) were identified and mean perceived wrongness of promiscuity was calculated for this group. Eleven participants in remote locations had no neighbors and were excluded, leaving *N* = 4,522. Mean neighbour number was 152.8 (*SD* = 129.5). There was a very weak but significant positive association between perceived wrongness of promiscuity and the mean value for nearest neighbors, *r*(4,520) = .09, *p* < .0001. Consequently, the spatially lagged (neighbors’) wrongness of promiscuity scores were used as a variable in subsequent analyses to control for spatial autocorrelation effects.

### Results

#### Individual-Level Analysis

Descriptive statistics and intercorrelations for individual-level variables are shown in Table 4. There was a significant positive association between perceived female economic dependence and wrongness of promiscuity with a small-to-moderate effect size (*r*[4,531] = .23, *p* < .001). A linear regression model was created with wrongness of promiscuity as the outcome variable and age, sex (males coded as 0, females as 1), perceived female economic dependence, religiosity, conservatism, and spatially lagged (neighbors’) wrongness of promiscuity as predictors. All predictors produced significant betas although that for neighbors’ wrongness of promiscuity was very weak (Table 5). When this analysis was conducted as a hierarchical regression, with perceived female economic dependence entered on the second step after the other five predictors had been entered on the first step, perceived female economic dependence explained an additional 2 % of variance in wrongness of promiscuity, Δ*R* ^2^ = .02, *F* = 102.10, *p* < .001.

**Table 4:** Intercorrelations, means, and *SD*s for Study 2 individual-level variables

Variable	1	2	3	4	5	6	7	8	*M*	*SD*	*N*
1. Age	–	.09***	.15***	.14***	.30***	.01	.04	.25***	30.71	11.05	2,206
2. Perceived FED	.04*	–	.17***	.26***	−.09***	−.17***	.11***	.29***	3.99	1.64	2,209
3. Religiosity	.10***	.14***	–	.42***	.02	−.11***	.11***	.42***	0.52	0.50	2,209
4. Conservatism	.08***	.23***	.34***	–	.05*	−.08**	.10***	.45***	2.44	1.07	2,209
5. Income	.38***	.07**	.07***	.12***	–	.46***	−.04	.02	2.84	2.21	2,209
6. Income ratio	.21***	.24***	.09**	.17***	.52***	–	−.04	−.12***	0.91	0.92	1,187
7. Neighbors	.03	.07***	.08***	.08***	−.02	.03	–	.11***	<.01	0.19	2,170
8. Wrongness of promiscuity	.13***	.21***	.39***	.39***	.07**	.19***	.07***	–	3.87	2.09	2,209
*M*	27.21	4.29	0.41	2.52	3.44	2.13	−0.01	3.31			
*SD*	9.07	1.43	0.49	1.05	2.73	2.31	0.20	1.86			
*N*	2,378	2,388	2,388	2,388	2,388	932	2,340	2,388			

Intercorrelations for males are below the diagonal, and intercorrelations for females are above the diagonal. Means and *SD*s for males are presented in the horizontal rows, and means and *SD*s for females are presented in the vertical columns. Neighbors = spatially-lagged neighbors’ wrongness of promiscuity; perceived FED = perceived female economic dependence. * *p* < .05, ** *p* < .01, *** *p* < .001

**Table 5:** Linear regression of wrongness of promiscuity on Study 2 predictors

	*β*	*t*	*p*
Age	.13	9.84	<.001
Sex (males = 0, females = 1)	.11	8.57	<.001
Perceived female economic dependence	.13	10.11	<.001
Religiosity	.26	18.60	<.001
Conservatism	.27	19.44	<.001
Spatially lagged (neighbors’) wrongness of promiscuity	.03	2.27	.023
	Overall: *N* = 4,497, total *R* = .54, Adj *R* ^2^ = .29, *p* < .001		

We also conducted supplementary analyses in which the model shown in Table 5 was considered when the participants were males only or females only and when the outcome variable was opposition to male promiscuity only or opposition to female promiscuity only. In all of these cases, the same general patterns were observed as those displayed in Table 5.

We next created two new regression models by adding personal income as a predictor to the model presented in Table 5 and analyzing results separately for male and female heterosexual participants. For females, income explained a small, but significant, amount of the variance in perceived wrongness of promiscuity, *β* = −.05, *t*(1,889) = −2.31, *p* = .021, but for males there was no significant association, *β* = −.03, *t*(2,156) = −1.49. Next, we created two more new regression models by replacing income with income ratio (ratio of own income to partner’s income) as a predictor in these models and analyzing results separately for male and female heterosexual participants involved in long-term relationships. For males, the amount of variance in wrongness of promiscuity explained by income ratio was significant in the predicted direction, but fairly small, *β* = .07, *t*(833) = 2.40, *p* = .017. For females, the amount of variance in wrongness of promiscuity explained by income ratio was nearly significant in the predicted direction and small, *β* = −.05, *t*(1,045) = −1.75, *p* = .08. In all four of the above models, perceived female economic dependence continued to explain significant variance in wrongness of promiscuity, after income or income ratio had been added as a predictor. Similar results were found in all four models, regardless of whether the outcome variable was opposition to male promiscuity or to female promiscuity.

#### State-Level Analysis

**Fig. 1:**
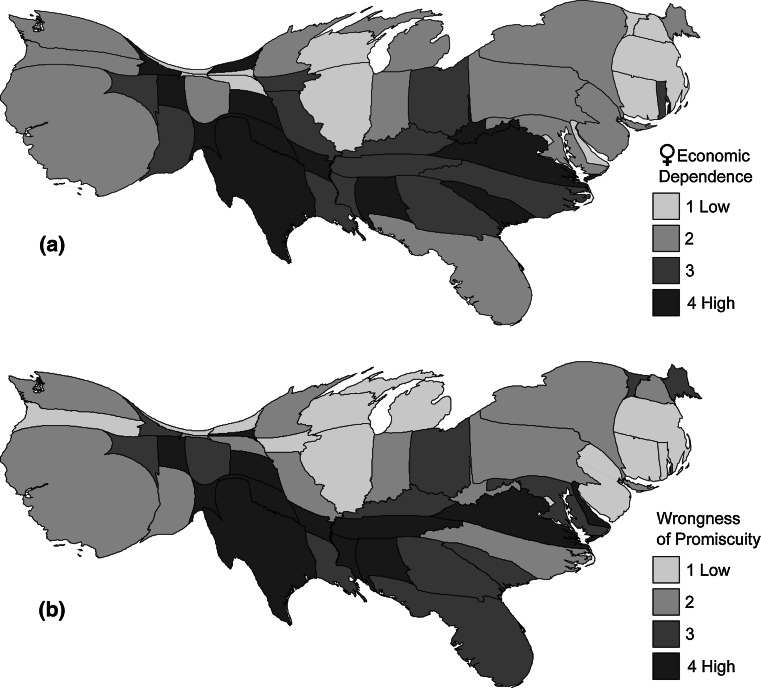
Cartograms of state mean scores for **a** perceived female economic dependence and **b** wrongness of promiscuity, showing quartile ranges, with state areas scaled to represent the number of participants (*N*) for each state. For state-level analyses, all tests were weighted by state *N*, so state areas represent each state’s relative influence in these analyses

Descriptive statistics and intercorrelations for state-level variables are shown in Table 6. For the state-level analysis, wrongness of promiscuity, perceived female economic dependence, religiosity, and conservatism were measured as within-state mean scores. To control for variation in sample sizes from each state, all correlational and regression results presented below were weighted by state *N*. (We also conducted an analysis in which, instead of weighting by *N*, we excluded states with samples sizes <20; using this alternative method did not change the direction or significance of the effects reported below). Correlations between wrongness of promiscuity and predictor variables tended to be much higher at the state level than they had been at the individual level, an illustration of the general principle that correlations between variables based on aggregated individual-level data will often be stronger than correlations between the same variables at the individual level (Ostroff, 1993). Cartograms in Fig. 1 display each state’s mean score on wrongness of promiscuity and perceived female economic dependence. (A cartogram is a map in which land area representations are manipulated according to some thematic variable; in the Fig. 1 cartograms, state areas are scaled to represent the number of participants from that state).

**Table 6:** Intercorrelations (weighted by *N*), means, and *SD*s for Study 2 state-level variables (within-state means)

Variable	2	3	4	5	6	7	8	9	*M*	*SD*	*N*
1. Perceived female economic dependence	.45**	.53***	−.49***	−.40**	−.23	−.18	.13	.66***	4.11	0.57	51
2. Religiosity	–	.73***	−.30*	−.25	−.15	−.03	−.30*	.64***	0.47	0.16	51
3. Conservatism	–	–	−.33*	−.29*	−.13	−.27	−.09	.73***	2.50	0.27	51
4. Median female income	–	–	–	.84***	.41**	.18	−.27	−.42**	24,462	4,273	51
5. Median male income	–	–	–	–	−.15	−.04	−.28*	−.39**	34,864	4,778	51
6. Female–male income ratio	–	–	–	–	–	.36*	.01	−.11	0.70	0.06	51
7. Welfare benefits	–	–	–	–	–	–	−.27	−.29*	653	202	51
8. Sex ratio	–	–	–	–	–	–	–	.03	1.02	0.03	51
9. Wrongness of promiscuity	–	–	–	–	–	–	–	–	3.59	0.56	51

* *p* < .05, ** *p* < .01, *** *p* < .0001

At the state level, wrongness of promiscuity was strongly positively related to perceived female economic dependence, *r*(49) = .66, *p* < .001, but unrelated to operational sex ratio, *r*(49) = .03. When wrongness of promiscuity was regressed on perceived female economic dependence, religiosity, and conservatism, betas were significant for perceived female economic dependence, *β* = .36, *t*(47) = 3.51, *p* = .001, and conservatism, *β* = .41, *t*(47) = 3.01, *p* = .004, but not for religiosity, *β* = .18, *t*(47) = 1.42. When this analysis was conducted as a hierarchical regression, with perceived female economic dependence entered on the second step after conservatism and religiosity had been entered on the first step, perceived female economic dependence explained an additional 9 % of variance in wrongness of promiscuity, Δ*R* ^*2*^ = .09, *F* = 12.33, *p* = .001.

In order to determine which state-level economic factors might be associated with the perception that females were economically dependent on males, we regressed perceived female economic dependence on state median female income, female–male income ratio, and welfare benefit level. Only median female income explained significant variance in perceived female economic dependence (Table 7) and, importantly, this negative association was not just a by-product of a negative relationship between perceived female economic dependence and income in general: when perceived female economic dependence was regressed on both median female income and median male income, only female, *β* = −.53, *t*(48) = −2.31, *p* = .025, but not male, *β* = .05, *t*(48) < 1, income was a significant predictor.

**Table 7:** Linear regression of perceived female economic dependence and wrongness of promiscuity (within-state means) on state-level economic predictors

	Perceived female economic dependence			Wrongness of promiscuity		
	*β*	*t*	*p*	*β*	*t*	*p*
Median female income	−.47	−3.41	.001	−.44	−3.12	.003
Female–male income ratio	−.01	<1	ns	.17	1.16	ns
Welfare benefits	−.09	<1	ns	−.28	−2.02	.049
	Overall: *N* = 51, total *R* = .50, Adj *R* ^2^ = .20, *p* = .003			Overall: *N* = 51, total *R* = .49, Adj *R* ^2^ = .20, *p* = .004		

*Note* Perceived female economic dependence = mean strength of perception that females in one’s social network depend economically on a male mate (by state). Wrongness of promiscuity = mean of anti-promiscuity morality for participants (by state)

**Fig. 2:**
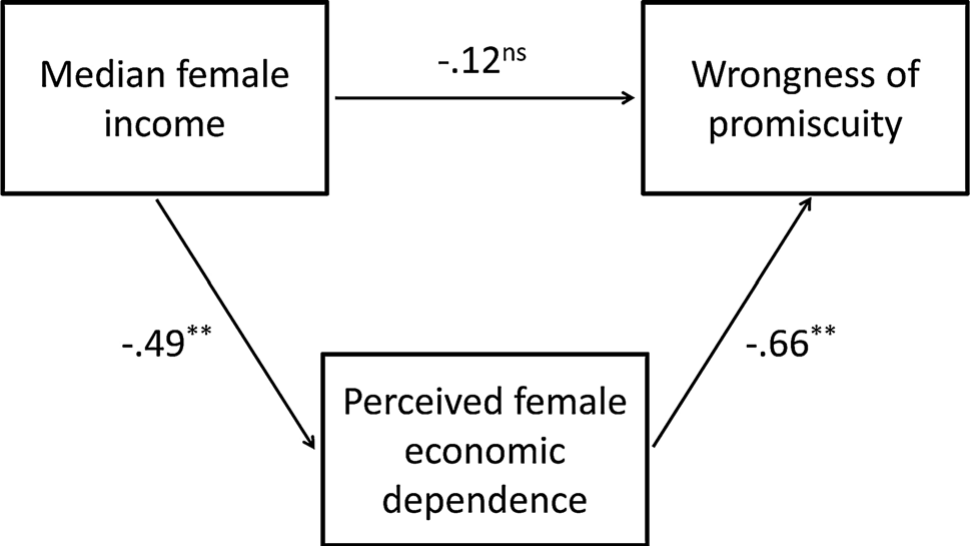
State level relationships between median female income, perceived female economic dependence and wrongness of promiscuity. Perceived female economic dependence = mean strength of perception that females in one’s social network depend economically on a male mate (by state). Wrongness of promiscuity = mean of anti-promiscuity morality for participants (by state). The significant negative association between median female income and wrongness of promiscuity, *r*(49) = −.42, *p* = .002, was fully mediated by perceived female economic dependence. Path coefficients are beta weights. **p < .001

When wrongness of promiscuity was regressed on these same three economic predictors (Table 7), there were significant negative associations with median female income and welfare benefit level. The latter effect only just reached significance and welfare benefit level explained no unique variance in perceived female economic dependence. Consequently, welfare benefit level was not included in a path model constructed to test the model assumptions that economic factors influence views on promiscuity via their influence on the extent to which females are perceived to depend economically on a male mate. As the only economic indicator that accounted for significant unique variance in both perceived female economic dependence and wrongness of promiscuity, median female income was included in this model. Figure 2 depicts this model, and displays the beta coefficients generated via standard multiple linear regression analyses. The significantly negative relationship between median female income and wrongness of promiscuity, *r*(49) = −.42, *p* = .002, was mediated fully by perceived female economic dependence.

### Discussion

Study 2 replicated the finding in Study 1 that perceived female economic dependence was a significant predictor of opposition to promiscuity, even after controlling for the effects of age, sex, religiosity, political conservatism, and also possible spatial associations between the anti-promiscuity views of geographical neighbors. Also replicated in Study 2 was the finding that, on average, women were more opposed to promiscuity than were men.

In addition to providing further evidence that perceived female economic dependence in one’s social network was a significant predictor of opposition to promiscuity, Study 2 results also suggested that anti-promiscuity views were strongest among those who were themselves involved in (or likely to become involved in) a relationship entailing high female economic dependence. Specifically, opposition to promiscuity was significantly lower among heterosexual females with higher incomes and significantly higher among heterosexual males who made more money relative to their partners. However, these personal income-related variables were generally weaker predictors of anti-promiscuity views than perceived female economic dependence in one’s social network.

Finally, Study 2 results indicated that perceived female economic dependence was a strong predictor of anti-promiscuity morality at the state level and that it accurately reflected female income levels within states. Unique variance in perceived female economic dependence was explained specifically by female income and not by female–male income ratio, male income or availability of welfare benefits. Female income was also negatively related to anti-promiscuity morality across states and this relationship was fully mediated by perceived female economic dependence. Contrary to the predicted result, however, a state’s operational sex ratio was unrelated to its level of anti-promiscuity morality.

## General Discussion

Both studies provided support for the female economic dependence theory of anti-promiscuity morality. According to this theory, in environments in which female economic dependence on a male mate is higher, both a woman and her mate have a greater interest in maximizing paternity certainty. Because promiscuity undermines paternity certainty, both men and women should be more opposed to promiscuity by both sexes in environments where there is greater female economic dependence on a male mate. Results from Studies 1 and 2 supported this theory, showing that anti-promiscuity morality was higher among men and women who perceived higher female economic dependence among women in their social network, even after controlling for relationships between one’s anti-promiscuity morality and one’s age, sex, religiosity, political conservatism, and the anti-promiscuity views of geographical neighbors. Furthermore, Study 2 suggested that, across states, perceived female economic dependence was related positively and uniquely to median female income (and not to any other state-level economic indicator) and this perception fully mediated the significantly negative relationship between female income and opposition to promiscuity across states.

Although the outcome variable in these studies was moral opposition to promiscuity rather than behavioral avoidance of promiscuity, these results were consistent with the view that people are flexible mating strategists, whose disposition towards one strategy versus another may vary facultatively according to conditions of one’s phenotype and one’s environment (Gangestad & Simpson, 2000). Considering that male parental investment is expected to often become more important in harsher environments, study results also complemented the finding that mean national sociosexuality levels correlated negatively with indicators of environmental hardship (Schmitt, 2005a). However, our results also clarified why a negative correlation between sociosexuality and hardship may not be observed in some environments. Schmitt (2005a) noted that some developmental-attachment theories (Belsky, Steinberg, & Draper, 1991; Chisholm, 1999) predict that hardship and sociosexuality will actually correlate positively rather than negatively. Importantly, these theories tend to emphasize problematic family relationships as the source of hardship. However, some kinds of problematic family relationships (e.g., father absence) may actually entail *reduced* female economic dependence on a male mate and so would not (according to the theory presented here) be expected to lead to lower promiscuity. In other words, the relevant predictor of promiscuity aversion may not be hardship in general, but rather female economic dependence on a male mate. Although females often do depend more on mates in harsher environments, in environments characterized by greater hardship, but not by greater female economic dependence on a mate (e.g., because men are unwilling or unable to provide key resources), we should not predict reduced promiscuity.

Results from both studies also suggested that women tend to be more opposed to promiscuity than men, which is consistent with the theory that men are in general more favorably disposed than women towards short-term mating (Schmitt, 2005a; Symons, 1979). Results from Study 2’s state-level analysis did not support the prediction, however, of higher anti-promiscuity morality in states with a higher proportion of men to women; instead, they suggested that such morality was unrelated to operational sex ratio. This result seems inconsistent with the finding (Schmitt, 2005a) that higher sociosexuality levels occur in nations with lower male–female sex ratios. More research is needed to evaluate the relationship between sex ratio and attitudes about mating strategies.

A particularly intriguing finding from Study 2 was that, although small amounts of significant variance in anti-promiscuity morality were predicted by personal income and ratio of own income to partner’s income (i.e., the extent to which one is currently, or is likely to become, involved in a relationship characterized by high female economic dependence), substantially more variance in this sentiment was predicted by perceived female economic dependence in one’s social network. These results suggest that moral views about promiscuity are influenced not just by one’s own calculations about the value of a promiscuous strategy to one’s self, but also, and more importantly, by the norms that prevail in one’s community about the value of promiscuity. This strategy of conforming to group cultural norms, however, should not be regarded as a “less biological” or “less individually-selected” behavior than that of selecting a personally-advantageous mating strategy. This is true for two reasons. First, adaptations for conforming to norms may have functioned to shield ancestral individuals from the negative fitness consequences of social ostracization (Ostrom, 2000). Second, anti-promiscuity norms are themselves proposed to be the outcome of individual-level fitness concerns related to paternal investment and paternity certainty.

### Why Focus on Female Economic Dependence Rather Than Religiosity and Conservatism?

Although perceived female economic dependence predicted more variance in anti-promiscuity morality than did personal circumstances, it was a weaker predictor at the individual level than religiosity or political conservatism. We argue, however, that female economic dependence has more conceptual utility when it comes to understanding the evolutionary logic of anti-promiscuity morality. Since restrictive sexual morality is a key element of most religious codes and politically conservative ideologies, individual-level associations between opposition to promiscuity and adherence to these belief structures are somewhat circular, by definition, and consequently not particularly informative. Humans are group-oriented and moralistic organisms and, as conservative and religious moral systems tend to oppose promiscuity, it is not surprising that members of these groups will also tend to oppose it. A more interesting issue is how these moral systems became so opposed to promiscuity in the first place. It is plausible that conservative and religious ideologies tend to oppose promiscuity because they themselves developed in environments with high female economic dependence on males. Regardless of the degree to which people who hold these beliefs continue to live in such environments, the beliefs may persist due to cultural evolutionary adaptive lag (Mesoudi, Whiten, & Laland, 2004), that is, because the environment has changed faster than the moral system. So although female economic dependence is contrasted with religiosity and conservatism in the above studies, these variables may actually be fundamentally related: religious and conservative moral systems may be anti-promiscuity because they themselves arose in environments where females depended heavily on male investment.

### Conclusion

Results of both studies were consistent with the theory that opposition to promiscuity arises in circumstances where paternity certainty is particularly important and suggest that such opposition will more likely emerge in environments in which women are more dependent economically on a male mate. Attempts to replicate these results in other cultures will be necessary in order to determine the robustness of this model under diverse social conditions. Further research will also be necessary to illuminate the psychological mechanisms that underlie the observed association between female economic dependence and opposition to promiscuity (e.g., the cues which shape individual perceptions of the local environment). One plausible mechanism is that people living in environments characterized by higher female dependence are more likely to learn about negative consequences associated with promiscuity (e.g., difficulties faced by parents and offspring in situations of high paternity uncertainty), a process which could generate a cultural opposition to promiscuity that is founded on biological concerns.

## Appendix A: Items Composing Perceived Female Economic Dependence, Wrongness of Promiscuity, and Religiosity in Study 1

1. Of the women I know who are in long-term heterosexual relationships, most rely financially on their male partner.
2. Of the women I know who are in long-term heterosexual relationships, most depend heavily on money contributed by their male partner.
3. Of the women I know who are in long-term heterosexual relationships, most do not rely much on financial assistance from their male partner.*
4. Of the women I know who are in long-term heterosexual relationships, most do not depend very heavily on money contributed by their male partner.*
5. Most women I know are financially independent from a male partner, or could be if they had to be.*
6. Most women I know depend heavily on the money of a male partner, or probably will at some point in their life.
7. Most women I know have a lot of financial independence, so they don’t need to rely on the money of a male partner.*
8. Most women I know will at some point in their life depend financially on a male partner.

*Reverse-coded

1. It is wrong for women to engage in promiscuous sex.
2. It is fine for a woman to have sex with a man she has just met, if they both want to.*
3. A woman should never have sex with a man she is not in love with.
4. There is nothing wrong with a woman being promiscuous.*
5. Promiscuous women are not worthy of much respect.
6. Women who sleep with lots of men deserve to be judged negatively.
7. It is wrong for men to engage in promiscuous sex.
8. It is fine for a man to have sex with a woman he has just met, if they both want to.*
9. A man should never have sex with a woman he is not in love with.
10. There is nothing wrong with a man being promiscuous.*
11. Promiscuous men are not worthy of much respect.
12. Men who sleep with lots of women deserve to be judged negatively.

*Reverse-coded

Religiosity (based on Kurzban et al., 2011). Item wordings provided by A. Dukes (personal communication, September 23, 2010):

1. How religious are you?

(Not at all religious, Somewhat religious, Very religious)

2. How spiritual are you?

(Not at all spiritual, Somewhat spiritual, Very spiritual)

3. Which of the following best describes how often you attend religious services?

(Never or almost never, A few times a year, About once a month, About every week, More than once a week)

4. Which of the following best describes how often you expect to attend religious services in the future?

(Never or almost never, A few times a year, About once a month, About every week, More than once a week)

5. Which of the following best describes how often you pray in private, on your own?

(Never or almost never, A few times a year, About once a month, About every week, Several times a week, About once a day, Several times a day)

## Appendix B: Items Composing Perceived Female Economic Dependence, Wrongness of Promiscuity, and Religiosity in Study 2

Our measures of perceived female economic dependence and wrongness of promiscuity in Study 2 were abbreviated, four-item versions on those used in Study 1. Principal components analysis of the items composing Study 1 variables was used to determine composition of Study 2 variables. For the eight items composing perceived female economic dependence in Study 1, this procedure produced one component with an eigenvalue greater than 1.0 (eigenvalue = 5.58, explaining 70.99 % of the variance in these items). The four items with the highest loadings on this component were selected to compose perceived female economic dependence in Study 2. These items (and their loading scores) were:

1. Of the women I know who are in long-term heterosexual relationships, most rely financially on their male partner. (.88)

2. Of the women I know who are in long-term heterosexual relationships, most depend heavily on money contributed by their male partner. (.88)

3. Of the women I know who are in long-term heterosexual relationships, most do not depend very heavily on money contributed by their male partner.* (−.87)

4. Most women I know depend heavily on the money of a male partner, or probably will at some point in their life. (.87)

*Reverse-coded

For the 12 items composing wrongness of promiscuity in Study 1, principal components analysis was conducting separately on the six items concerning male promiscuity and the six items concerning female promiscuity. This was done so that the two highest-loading items related to promiscuity in each sex could be incorporated into the Study 2 variable, and an equal balance between male and female promiscuity could be maintained. For the six items related to female promiscuity, this procedure yielded one component with an eigenvalue greater than 1.0 (eigenvalue = 4.32, explaining 71.93 % of the variance in these items). These two items (and their loading scores) were:

1. It is wrong for women to engage in promiscuous sex. (.90)
2. There is nothing wrong with a woman being promiscuous.* (−.89)

For the six items related to male promiscuity, this procedure yielded one component with an eigenvalue greater than 1.0 (eigenvalue = 4.51, explaining 75.19 % of the variance in these items). These two items (and their loading scores) were:

3. It is wrong for men to engage in promiscuous sex. (.90)
4. There is nothing wrong with a man being promiscuous.* (−.90)

*Reverse-coded

Thus, the two items related to female promiscuity were worded in the same ways as the two items related to male promiscuity.

Our Study 2 religiosity measure was an abbreviated version of the Study 1 measure; in that measure, the item that most directly measured religiosity, “How religious are you?”, was highly correlated with the entire measure (r_s_ = .86), so we used this item as our Study 2 religiosity measure. Responses were on a three point scale (“not at all”, “somewhat”, and “very”); we combined the “somewhat” and “very” categories to convert religiosity into a binary nominal variable (“non-religious” or “religious”) for use in regression analyses.
